# Lung Adenocarcinoma in a Patient with Plasmacytoma

**DOI:** 10.1155/2013/726437

**Published:** 2013-12-22

**Authors:** Atsunori Hiasa, Kazunori Nakase, Kazuo Fukutome, Hideki Nomura, Setsuko Ueno, Toshiro Mizuno, Naoyuki Katayama, Toshiaki Takeuchi

**Affiliations:** ^1^Department of Internal Medicine, Toyama Hospital, Tsu, Mie 514-0043, Japan; ^2^Cancer Center, Mie University Hospital, Tsu, Mie 514-8507, Japan; ^3^Department of Hematology and Oncology, Mie University Hospital, Tsu, Mie 514-8507, Japan

## Abstract

An increased risk of second malignancy is well recognized in patients treated for plasma cell neoplasms. However, second solid tumor is very rare in such patients. We report a case of a 68-year-old woman with plasmacytoma who developed lung adenocarcinoma.

## 1. Introduction

The development of effective treatment for hematological malignancy has resulted in a large number of long-term survivors. As a result of survival elongation, second malignancy has been increasingly recognized [1, 2]. In patients with multiple myeloma, elevated risks of acute myeloid leukemia (AML) and myelodysplastic syndrome (MDS) have been described for over 4 decades. On the other hand, second solid malignancy is very rare in such patients [3, 4]. We present a case of lung adenocarcinoma in a patient with plasmacytoma and discuss the pathogenesis of the occurrence of lung cancer (LC) in this patient.

## 2.   Case Report

**Figure 1:**
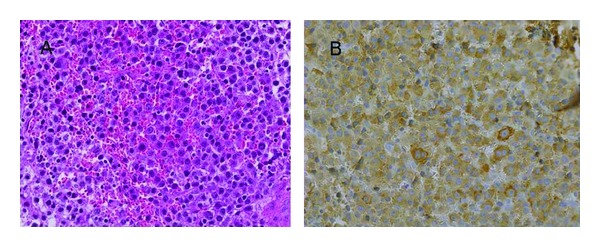
Histological features of the tumor in the 1st lumber vertebra. (A) Dense infiltrates of monomorphic plasma cells were seen (hematoxylin-eosin stain). (B) Plasma cells were positive for immunoglobulin *λ* light chain (immunohistochemical stain).

**Figure 2:**
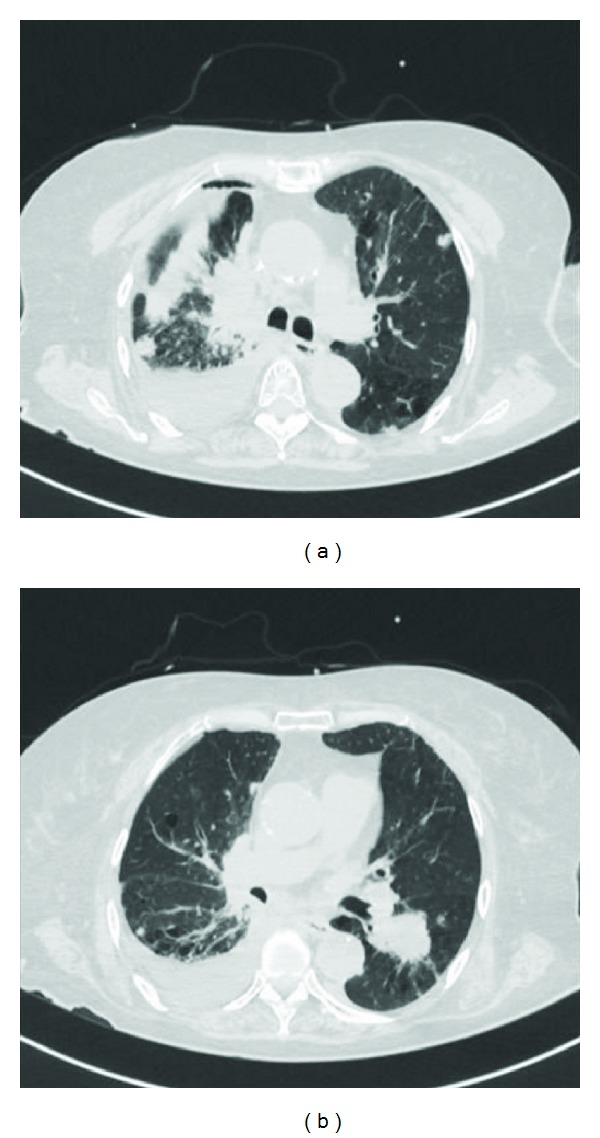
Chest CT scan demonstrated multiple tumors in the bilateral lungs and a right-sided pleural effusion.

**Figure 3:**
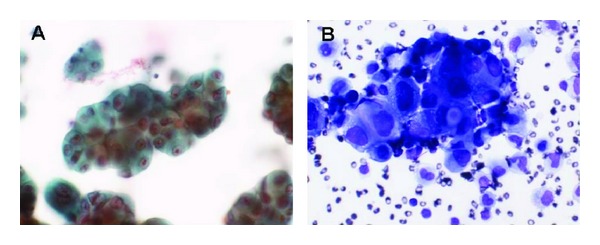
Adenocarcinoma cells in the pleural effusion. (A) Papanicolaou stain. (B) May-Giemsa stain.

A 68-year-old woman, who had been a heavy smoker (40 cigarettes/day for 45 years), was admitted to the Mie university hospital with lumbago and progressive lower body paralysis in May 2001. She received an emergent operation because of the compression of the spinal cord by the decompression fracture of the 1st lumbar vertebra. A diffuse accumulation of plasma cells was disclosed by the pathological examination of the bone tissue taken at that time (Figure 1). Although serum immunoglobulin (Ig) levels were not increased (IgG 1205 mg/dL), serum immunoelectrophoresis showed a monoclonal IgG-*λ* bow. No increased number of plasma cells was observed in the bone marrow from the sternum and the ilium. Bone scintigraphy showed an increased uptake in the 1st lumbar vertebra and the left humerus. She was diagnosed with multiple plasmacytoma and received local radiation therapy (RT) for their lesions (35 Gy each). After a while, new lesions continuously appeared, and all of those were treated with local RTs: the sternum (40 Gy) in December 2001, the right femur (35 Gy) in February 2002, the right sacroiliac articulation (35 Gy) in April 2002, the right humerus (30 Gy) in December 2002, the left breast (50 Gy) in June 2003, the left jaw (40 Gy) in April 2004, the right 11th rib (35 Gy) in May 2004, the left kidney surrounding lesion (40 Gy) and the anterior bladder lesion (40 Gy) in May 2006, and the mediastinum (40 Gy) and the right fibula (40 Gy) in September 2006. Lumpectomy was performed for the left breast lesion, which was confirmed as plasmacytoma. Chemotherapy with melphalan (6 mg/day) and prednisolone (40 mg/day) for 4 days every 4–6 weeks was also initiated in November 2006. After 37th courses of this regimen, she was admitted to our hospital because of dyspnea in January 2011. Chest computer tomography (CT) scan revealed multiple tumors in the bilateral lungs and a right-sided pleural effusion (Figure 2). Carcinoembryonic antigen was elevated (47.0 ng/mL). A chest tube was inserted into her right pleural cavity and the effusion fluid was drawn out. Cytology of this fluid revealed adenocarcinoma (Figure 3). Mutations of epidermal growth factor receptor gene in these cancer cells were not detected. In March 2011, she died of respiratory failure irrespective of various supportive therapies. Autopsy was not done.

## 3. Discussion

The treatment of plasma cell malignancy has progressed in the past 4 decades. These advances, however, have been accompanied by a concern for second malignancies. The development of AML and MDS has been described in patients with multiple myeloma treated with an alkylating agent, melphalan, and/or an immunomodulatory drug, lenalidomide [1, 2]. Our case developed LC following chemotherapy with melphalan for about 4 years. However, the incidence and diversity of solid malignancy in myeloma patients has been reported to be similar to those of normal persons of the same age [3, 4]. While in patients with Hodgkin's lymphoma (HL), Travis et al. [5] reported that the risk of LC significantly elevated within 1–4 years after treatment with alkylating agents such as mechlorethamine and procarbazine. These observations suggest that the alkylating agent used for myeloma seems to be unrelated to the pathogenesis of second solid tumors.

On the other hand, RT may also have a potential role in the development of therapy-induced malignancies. RT is the most effective standard treatment of plasmacytoma [6, 7]. In patents with HL, many studies have noted the largest risk for LC as late effects of successful RT [5, 8–10]. As for the total dose of RT given to the patient, more than 9 Gy was described to be related to the elevated risk of LC in HL patients [8, 11]. An excess risk of LC following RT was suggested to begin 5 years after treatment [5]. Furthermore, some studies have reported a higher risk of LC after HL in smokers than in nonsmokers [8, 11]. Even in breast cancer, postmastectomy RT was also demonstrated to significantly increase the risk of LC [12–15]. Our patient was treated with RTs (more than 50 Gy) to the left breast and mediastinum about 5 years ago. Some lung fields were involved by such radiation exposures. Moreover, she was a heavy smoker for 45 years. Previous studies have identified the joint effects of cigarette smoking and postmastectomy RT on LC risk. Therefore, RT coupled with smoking might be also associated with the development of LC in our case. Concerning the morphology of LC after RT, squamous cell carcinoma was reported to be the most common subtype [14]. However, our case revealed adenocarcinoma. Kaufman et al. [15] described that smoking and postmastectomy RT were associated with all histologic types of LC. Accordingly, no particular tendency in the LC subtype may be found in patients like our case.

Unlike multiple myeloma, as RT could be used frequently for plasmacytoma, such a case may have a possibility of the development of RT-induced malignancy. In this case, although the coincidental coexistence of double cancers could not be completely denied, we should pay attention to the occurrence of LC in patients with plasmacytoma receiving chest involved RTs as well as patients with HL or breast cancer, especially in heavy smokers.

## References

[1] Thomas A, Mailankody S, Korde N, Kristinsson SY, Turesson I, Landgren O (2012). Second malignancies after multiple myeloma: from 1960s to 2010s. *Blood*.

[2] Nakase K, Tsuji K, Hasegawa M (1995). Acute myelomonocytic leukemia in a patient with multiple myeloma: evidence for different clonal origin. *Internal Medicine*.

[3] Stegman R, Alexanian R (1979). Solid tumors in multiple myeloma. *Annals of Internal Medicine*.

[4] The Finnish Leukaemia Group (2000). Acute leukaemia and other secondary neoplasms in patients treated with conventional chemotherapy for multiple myeloma: a Finnish Leukaemia Group study. *European Journal of Haematology*.

[5] Travis LB, Gospodarowicz M, Curtis RE (2002). Lung cancer following chemotherapy and radiotherapy for Hodgkin’s disease. *Journal of the National Cancer Institute*.

[6] Mayr NA, Wen BC, Hussey DH (1990). The role of radiation therapy in the treatment of solitary plasmacytomas. *Radiotherapy and Oncology*.

[7] Jyothirmayi R, Gangadharan VP, Nair MK, Rajan B (1997). Radiotherapy in the treatment of solitary plasmacytoma. *British Journal of Radiology*.

[8] van Leeuwen FE, Klokman WJ, Stovall M (1995). Roles of radiotherapy and smoking in lung cancer following Hodgkin’s disease. *Journal of the National Cancer Institute*.

[9] Swerdlow AJ, Schoemaker MJ, Allerton R (2001). Lung cancer after Hodgkin’s disease: a nested case-control study of the relation to treatment. *Journal of Clinical Oncology*.

[10] Lorigan P, Radford J, Howell A, Thatcher N (2005). Lung cancer after treatment for Hodgkin’s lymphoma: a systematic review. *Lancet Oncology*.

[11] Amadori D, Ronconi S (2005). Secondary lung tumors in hematological patients. *Seminars in Respiratory and Critical Care Medicine*.

[12] Zablotska LB, Neugut AI (2003). Lung carcinoma after radiation therapy in women treated with lumpectomy or mastectomy for primary breast carcinoma. *Cancer*.

[13] Ford MB, Sigurdson AJ, Petrulis ES (2003). Effects of smoking and radiotherapy on lung carcinoma in breast carcinoma survivors. *Cancer*.

[14] Prochazka M, Hall P, Gagliardi G (2005). Ionizing radiation and tobacco use increases the risk of a subsequent lung carcinoma in women with breast cancer: case-only design. *Journal of Clinical Oncology*.

[15] Kaufman EL, Jacobson JS, Hershman DL, Desai M, Neugut AI (2008). Effect of breast cancer radiotherapy and cigarette smoking on risk of second primary lung cancer. *Journal of Clinical Oncology*.

